# Identification of Novel Gene Regulatory Networks for Dystrophin Protein in Vascular Smooth Muscle Cells by Single-Nuclear Transcriptome Analysis

**DOI:** 10.3390/cells12060892

**Published:** 2023-03-14

**Authors:** Yan Shen, Il-man Kim, Yaoliang Tang

**Affiliations:** 1 Medical College of Georgia, Augusta University, Augusta, GA 30912, USA; 2 Department of Anatomy, Cell Biology and Physiology, School of Medicine, Indiana University, Indianapolis, IN 46202, USA

**Keywords:** blood pressure, dystrophin, Duchenne muscular dystrophy, KCNQ5, and single nuclear RNA sequencing

## Abstract

Duchenne muscular dystrophy is an X-linked recessive disease caused by mutations in dystrophin proteins that lead to heart failure and respiratory failure. Dystrophin (*DMD*) is not only expressed in cardiomyocytes and skeletal muscle cells, but also in vascular smooth muscle cells (VSMCs). Patients with DMD have been reported to have hypotension. Single nuclear RNA sequencing (snRNA-seq) is a state-of-the-art technology capable of identifying niche-specific gene programs of tissue-specific cell subpopulations. To determine whether *DMD* mutation alters blood pressure, we compared systolic, diastolic, and mean blood pressure levels in mdx mice (a mouse model of DMD carrying a nonsense mutation in *DMD* gene) and the wide-type control mice. We found that mdx mice showed significantly lower systolic, diastolic, and mean blood pressure than control mice. To understand how *DMD* mutation changes gene expression profiles from VSMCs, we analyzed an snRNA-seq dataset from the muscle nucleus of *DMD* mutant (*DMD*^mut^) mice and control (Ctrl) mice. Gene Ontology (GO) enrichment analysis revealed that the most significantly activated pathways in *DMD*^mut^-VSMCs are involved in ion channel function (potassium channel activity, cation channel complex, and cation channel activity). Notably, we discovered that the *DMD*^mut^-VSMCs showed significantly upregulated expression of KCNQ5 and RYR2, whereas the most suppressed pathways were transmembrane transporter activity (such as anion transmembrane transporter activity, inorganic anion transmembrane transporter activity, import into cell, and import across plasma membrane). Moreover, we analyzed metabolic pathways from the Kyoto Encyclopedia of Genes and Genomes (KEGG) using “scMetabolism” R package. *DMD*^mut^-VSMCs exhibited dysregulation of pyruvate metabolism and nuclear acid metabolism. In conclusion, via the application of snRNA-seq, we (for the first time) identify the potential molecular regulation by *DMD* in the upregulation of the expression of KCNQ5 genes in VSMCs, which helps us to understand the mechanism of hypotension in DMD patients. Our study potentially offers new possibilities for therapeutic interventions in systemic hypotension in DMD patients with pharmacological inhibition of KCNQ5.

## 1. Introduction

Duchenne muscular dystrophy is an X-linked genetic disorder caused by the absence of dystrophin (*DMD*), a gene that encodes a 427-kD protein that links the sarcomere and the extracellular matrix [1]. *DMD* functions to stabilize *DMD*-associated glycoprotein complex (DGC) during cyclic muscle contraction and relaxation [2]. DMD patients have symptoms in early childhood, and gradually lose mobility during adolescence due to progressive muscle cell atrophy, which is replaced by fibrotic tissue. Patients typically die from heart or pulmonary failure in their third decade [3]. The most common cardiovascular manifestations of DMD are dilated cardiomyopathy, arrhythmias, and congestive heart failure [4]. However, several case reports show that *DMD* deficiency impacts blood pressure in patients with Duchenne muscular dystrophy. Ryan TD et al. [5] compared central blood pressure in 43 patients with DMD and found that central systolic blood pressure was lower in patients with DMD when compared to normal control. Moreover, Li J. et al. [6] reported that a 15-year-old boy with Duchenne muscular dystrophy underwent hypotension following the administration of the angiotensin-receptor/neprilysin inhibitor sacubitril/valsartan, indicating that the boy could not even tolerate a small dose of the anti-hypertension drug. In this study, we compared diastolic, systolic, and mean blood pressure levels in *DMD*^mut^ and control mice, and found that *DMD*^mut^ mice had lower systolic, diastolic, and mean blood pressure than wild-type controls.

The molecular mechanisms by which *DMD* mutation contributes to hypotension remains unknown. To identify *DMD*-regulated gene networks in vascular smooth muscle cells (VSMCs), we systematically compared gene expression profiles of VSMCs from *DMD*^mut^ and control mice using state-of-the-art single nuclear RNA sequencing (snRNA-seq), which is a revolutionary technique that allows unbiased identification of gene expression heterogeneity at the cellular level. We identified major differentially expressed genes (DEGs), including KCNQ5 and RyR2. The functional enrichment analysis illuminated that the majority of DEGs were enriched in potassium channel activity and cation channel complex. Moreover, to quantify single-cell metabolic activity, we performed scMetabolism analysis, and found that *DMD*^mut^-VSMCs exhibited dysregulation of numerous energy metabolic pathways, including pyruvate metabolism, amino acid metabolism, and nuclei acid metabolism. 

## 2. Materials and Methods

### 2.1. Immunofluorescent Staining

To detect dystrophin expression in hearts, we removed hearts from 12-month-old male D2.B10-*DMD*Mdx/J mice (*DMD*^mut^) and control DBA/2J mice (Control) (The Jackson Laboratory, ME, USA). Tissues were embedded in OCT compound, snap frozen, cut into 5-μm sections, fixed with 4% paraformaldehyde, blocked with 5% goat serum for 1 h, and immunostained with rabbit anti-Myh11 (1:100; Abcam, Waltham, MA, USA) antibody and Alex488-conjugated anti-Dystrophin antibody (1:50, Santa Cruz). Primary antibodies were resolved via secondary staining with Alexa555-conjugated goat-anti-rabbit antibody (1:400, Thermo Fisher, Waltham, MA, USA). Nuclei were counterstained with DAPI (Vector Laboratories, Newark, CA, USA). All imaging was performed on a Leica confocal microscope (Leica Microsystems Inc. Deerfield, IL, USA).

### 2.2. Assessment of Blood Pressure via Tail-Cuff

We examined blood pressure in 6-month-old male *DMD*^mut^ and control mice by using a volume-pressure recording tail-sleeve blood pressure measurement system (CODA-HT2 High Throughput, Kent Scientific Corporation, Torrington, CT, USA). Each mouse was placed in a restrainer with a black nose cone to simulate a dark environment as a reduction of stress. The tails of mice were placed on a surgical platform at a temperature of 32–35 °C. The tail-cuff was gently placed over the tail near the base of the tail. Acclimatization was performed with the restraint and tail sleeve inflated for 30 min each day for 2 days prior to experimental measurements. Blood pressure measurements on conscious mice were performed in a random and blinded manner. Each measurement started 15 min after acclimation for a total of 45 measurements over the 3 sessions. Data were collected and saved for later analysis using Coda software (Kent Scientific Corporation, Torrington, CT, USA). The first 5 measurements were not used for analysis to avoid the effect of adaption of the animal to the tail-cuff inflations. Unreliable recordings were eliminated. Manual elimination was performed only when the animal showed excessive movements generating artificial signals. 

### 2.3. Single-Cell RNA-Sequencing Datasets

The snRNA-seq data (barcodes, features, and matrix of gene expression) were downloaded from the NCBI Gene Expression Omnibus (GEO) public database (GSE156498) [7].

### 2.4. SnRNA-Seq Data Analysis

Seurat R package (V4.2) was used for downstream analytic procedures. Cells with extreme feature counts (<200 or >2500) and >20% reads with mitochondrial alignment were removed. Subsequently, we performed data normalization, high-variance feature identification, data scaling, and principal component analysis (PCA) using Seurat’s classic workflow. Then, the Harmony algorithm was used to correct the batch effects among samples. Next, dimensional reduction was performed using Uniform Manifold Approximation and Projection (UMAP) with the parameter “reduction” set as “harmony”. Seurat’s “FindNeighbors” and “FindClusters” functions were applied to the cell clustering analysis. VSMCs were annotated according to the contractile smooth-muscle lineage marker gene Myosin heavy chain 11 (MYH11) or pan-smooth muscle cell marker α-smooth muscle actin (ACTA2), and the VSMC cluster was used for downstream analysis. The VSMC cluster was further re-clustered to generate a cluster subset (0–4). To identify differentially expressed genes (DEGs) between *DMD*^mut^ VSMCs and control VSMCs, “FindMarkers” function under the default Wilcoxon rank sum test was applied to identify DEGs with avg_log2FC > 1 and *p*_val_adj < 0.05 as significant differential abundance. Volcano plots were generated using the R package “EnhancedVolcano” (V1.14). 

### 2.5. Gene Ontology (GO) and GSEA Enrichment Analysis

We applied the gseGO function from R/Bioconductor “clusterProfiler package” (V4.4.4) and org.Mmu.eg.db (V3.15) to perform gene ontology (GO) pathway enrichment analysis and Gene Set Enrichment Analysis (GSEA) of DEGs with default parameters. The terms with *p*-values < 0.05 identified from GO pathway enrichment analysis were considered significant. GO enrichment analyses were visualized as bubble plots, and the network of most enriched terms was visualized by the cnetplot function. 

### 2.6. Single-Cell Metabolic Analysis

To discern the metabolic difference between *DMD*^mut^ and control VSMCs in snRNA-seq datasets, we applied the “scMetabolism” package to quantify the metabolic activities of individual VSMCs within *DMD*^mut^ versus control VSMCs. Specifically, the method was set to “VISION”, and analyzed using the Kyoto Encyclopedia of Genes and Genomes (KEGG) metabolic gene sets.

### 2.7. Statistical Analysis

Statistical analyses were performed using R language (R version 4.2). For blood pressure analysis, values were expressed as mean ± standard error of mean (SEM). Student’s t-test was used to compare two groups using GraphPad Prism (V9), and *p* < 0.05 was considered statistically significant.

## 3. Results

### 3.1. Expression of DMD in Vascular Smooth Muscle Cells

**Figure 1:**
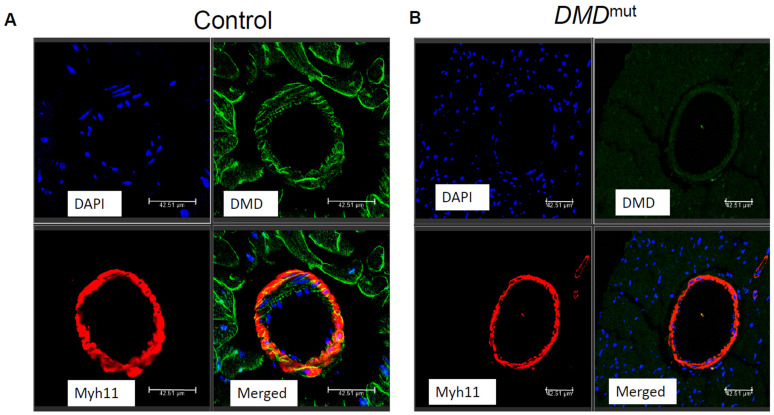
*DMD* expression in vascular smooth muscle cells from cross section in the hearts of WT and Mdx mice. (**A**) *DMD* protein expression in the WT heart. (**B**) *DMD* protein expression in the *DMD*^mut^ heart.

To examine whether vascular smooth muscle cells express *DMD* protein, we performed double immunostaining for mature vascular smooth muscle cell marker Myh11 and *DMD*. We observed that the *DMD* expression was localized to the mature vascular smooth muscle cells (co-localized with Myh11) in control hearts, but not in the *DMD*^mut^ hearts (Figure 1A,B).

### 3.2. DMD^mut^ Mice Have Lower Blood Pressure Compared to Control Mice

**Figure 2:**
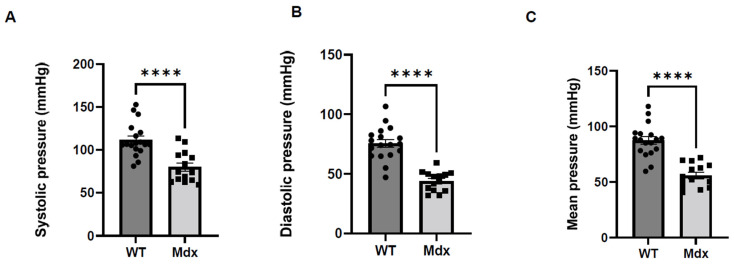
Blood pressure was lower in *DMD*^mut^ (Mdx) versus wild-type mice. (**A**) Systolic pressure (mmHg), (**B**) diastolic BP (mmHg), and (**C**) mean pressure (mmHg) were collected using a Coda BP system in WT and Mdx mice (**** *p* < 0.0001, *n* = 18 for WT mice, *n* = 14 for *DMD*^mut^ mice).

We compared systolic and diastolic blood pressure between control and *DMD*^mut^ mice at the age of 6 months. We found that there was significantly lower systolic, diastolic, and mean blood pressure in *DMD*^mut^ mice compared to control mice (Figure 2A–C).

### 3.3. Identification of VSMC Clusters in DMD^mut^ and Control Muscles through snRNA-Seq Analysis

**Figure 3:**
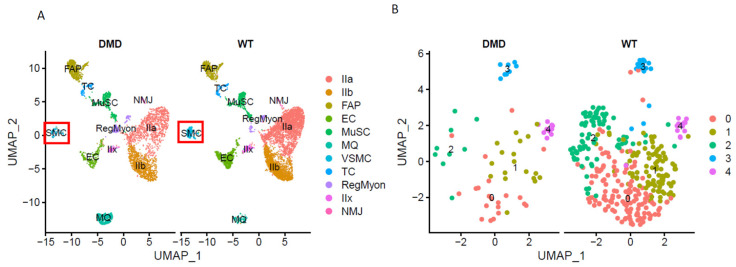
Single-nuclei RNA sequence (snRNA-seq) analyses. (**A**) Split view of UMAP plot representation of muscle-derived nuclei from the *DMD*^mut^ and WT mouse samples. Each major cell type was represented with a different color. A red square indicates VSMC subsets from muscle nuclei. (**B**) Split view of UMAP plot representation of the VSMCs from *DMD*^mut^ and WT mouse samples.

GSE156498 contains two samples; one was from a *DMD*^mut^ muscle nucleus and the other was from a control muscle nucleus. After a rigorous quality control procedure, potential batch effects between samples processed in different batches were eliminated by using the RunHarmony function in the Harmony package before performing unsupervised graph-based clustering analysis. Subsequently, we segmented VSMCs based on the expression of a typical VSMC marker (MYH11). Ultimately, we obtained a sparse matrix containing 336 VSMCs from 6,409 muscle nuclei (~5.2%) and 66 VSMCs from 3937 *DMD*^mut^ muscle nuclei (~1.7%) (Figure 3A), suggesting that control muscles contain more VSMCs than *DMD*^mut^ muscles. We then performed unbiased clustering of transcriptional profiles of VSMCs in each sample and obtained a total of 5 subclusters (clusters 0–4) through UMAP analysis (Figure 3B).

### 3.4. Differential Gene Expression and Functional Enrichment Analysis of VSMCs in DMD^mut^ versus Control Muscles

**Figure 4:**
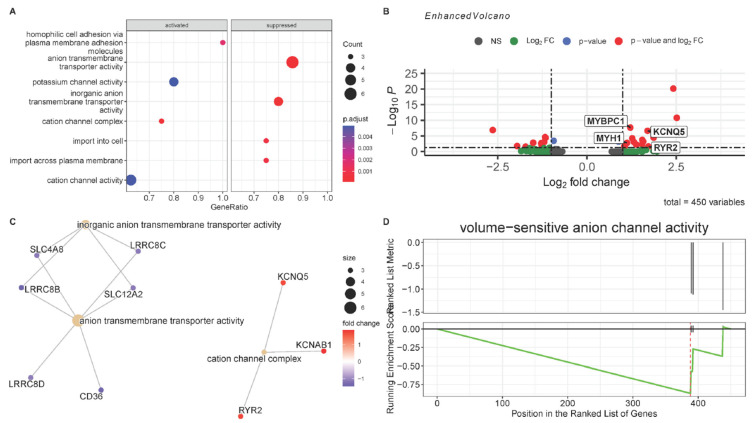
Functional enrichment analysis of VSMCs and VSMC-specific expression change of *DMD*^mut^ and WT muscles. (**A**) Dot plot representing the top 4 gene ontologies (GOs) with the largest gene ratios in order of gene ratio. The color gradient of dots represents the adjusted P values, while the size represents the number of genes in the significant DEG list associated with the GO term. (**B**) Volcano plots showing the adjusted P values and the log2 fold change (FC) value of genes in the VSMCs from *DMD*^mut^ and WT muscles. DEGs, indicated by the red dots, were identified as genes with an adjusted *p*-value of ≤ 0.05 and |log2FC| > 0.26. KCNQ5, RyR2, MYH1, and MYBPC1 were indicated by the square. (**C**) The cnetplot depicts the linkages of genes and GO terms as a network, which is helpful to see which genes are involved in enriched pathways. (**D**) GSEA Plot of the Running Enrichment Score (green line) for a gene set as the analysis walks down the ranked gene list, including the location of the maximum enrichment score (the red line). The black lines in the Running Enrichment Score show where the members of the gene set appear in the ranked list of genes, indicating the leading edge subset.

To investigate the differentially expressed genes (DEGs) between VSMCs residing in *DMD*^mut^ versus control muscles, we performed a gene ontology (GO) enrichment analysis using the clusterProfiler package. The GO term analysis showed that the most significantly activated pathways in *DMD*^mut^-VSMCs were involved in ion channel function (potassium channel activity, cation channel complex, and cation channel activity) (Figure 4A). Notably, we discovered that the *DMD*^mut^-VSMCs showed significantly upregulated expression of potassium voltage-gated channel subfamily Q member 5 (KCNQ5), ryanodine receptor 2 (RYR2), myosin heavy chain isoform 1 (MYH1), and the slow-twitch skeletal muscle myosin binding protein C1 (MYBPC1), et al. (Figure 4B). KCNQ5 was expressed in the brain, skeletal muscle, and blood vessels [8]. The Kv7.5 potassium channel subunit, the product of KCNQ5, had an influence on vascular reactivity. In addition, RyR2 had an essential function in systemic blood pressure control and vascular adaptive responses to pressure [9]. These findings emphasized the key role of activation of channel pathways in *DMD*^mut^-VSMCs.

The GO term analysis also revealed that the most significantly suppressed pathways in *DMD*^mut^-VSMCs were mainly enriched in transmembrane transporter activity (such as anion transmembrane transporter activity, inorganic anion transmembrane transporter activity, import into cell, and import across plasma membrane) (Figure 4A). The significant downregulated genes included DTX3, AHNAK, SRRM2, GUCY1A2, PID1, SON, PDE7B, etc. (Figure 4B). PDE7B (Phosphodiesterase 7B) hydrolyzed the second messenger cAMP, a key regulator of many important physiological process [10]. DTX3 was reported to promote the degradation of Notch2. SRRM2 is a serine/arginine-rich protein involved in RNA splicing. AHNAK plays an important scaffolding function connecting Erk and Rac activation in PDGF-dependent aortic smooth cell migration [11]. To explore the complex association between the identified top GO groups, we used the cnetplot function, which depicts the linkage of the most-enriched GO terms and the genes involved in these terms as a network, which allows visualization of the genes involved in the enriched pathways and genes that may belong to annotation categories (Figure 4C), which showed an activated cation channel complex network, which included KCNQ5, KCNAB1, RYR2, while the anion transmembrane transporter activity network and inorganic anion transmembrane transporter activity network, which included SLC4A8, SLC12A2, CD36, LRRC8B-D, were suppressed. Our GSEA results indicated that the gene set associated with volume-sensitive anion channel activity was significantly suppressed in *DMD*^mut^ VSMCs compared to control VSMCs (Figure 4D).

### 3.5. Differential Metabolic Pathways of VSMCs within DMD^mut^ and the Control Muscle

**Figure 5:**
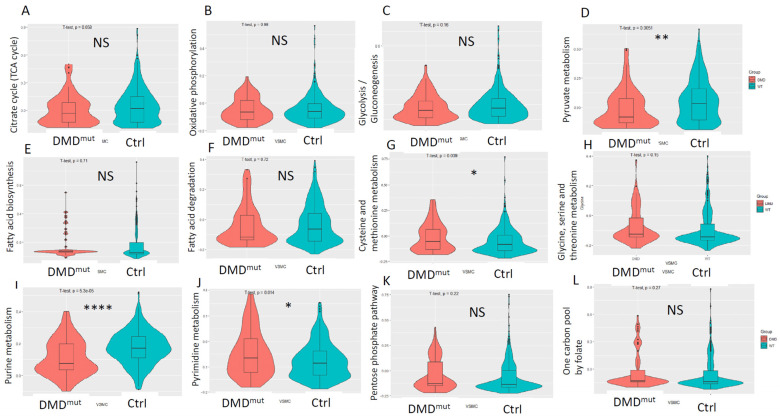
Violin plot illustrations of the metabolic pathway analyses performed by scMetabolism package for VSMCs from *DMD*^mut^ and control muscles. Violin plots comparing citrate cycle (TCA cycle) (**A**), oxidative phosphorylation (**B**), glycolysis/gluconeogenesis (**C**), pyruvate metabolism (**D**), fatty acid biosynthesis (**E**), fatty acid degradation (**F**), cysteine and methionine metabolism (**G**), glycine, serine and threonine metabolism (**H**), purine metabolism (**I**), pyrimidine metabolism (**J**), pentose phosphate pathway (**K**), and one carbon pool by folate (**L**) (NS: no significant difference, * *p* < 0.05, ** *p* < 0.01, **** *p* < 0.0001, *n* = 66 for *DMD*^mut^ VSMCs, *n* = 336 for control VSMCs).

To investigate the metabolic change in VSMCs after *DMD* mutation, we investigated the metabolic landscape using the “scMetabolism” package, which focuses on a comprehensive collection of metabolic pathways. We first filtered the metabolic pathways and compared the activity between VSMCs within *DMD*^mut^ and control muscles. The results suggested a significant difference in the enrichment of multiple metabolic pathways: (i) There was no statistical difference in activities of oxidative citric acid cycle (TCA cycle) and phosphorylation (OXPHOS) in VSMCs from *DMD*^mut^ versus control muscles; (ii) there was no statistical difference in activity of glycolysis/gluconeogenesis, but there was significantly reduced activity of pyruvate metabolism in VSMCs from *DMD*^mut^ versus control muscles; (iii) there was no statistical difference in activity of fatty acid biosynthesis and the fatty acid degradation in VSMCs from *DMD*^mut^ muscles versus control muscles; (iv) as for amino acid metabolic pathways, the activity of cysteine and methionine metabolism was slightly upregulated in VSMCs from *DMD*^mut^ versus control muscles, but there was no statistical difference in activity of glycine, serine, and threonine metabolism in VSMCs from *DMD*^mut^ versus control muscles; (v) compared to VSMCs in control muscles, VSMCs from *DMD*^mut^ muscles exhibited significantly increased pyrimidine metabolic activity and significantly decreased purine metabolism; and (vi) there was no statistical difference in activity of both one carbon pool by folate, and pentose phosphate pathway activity were increased in VSMCs from *DMD*^mut^ versus control muscles (Figure 5A–L).

### 3.6. DMD Mutation Reduces the Expression of Contractile Gene Markers of VSMCs

**Figure 6:**
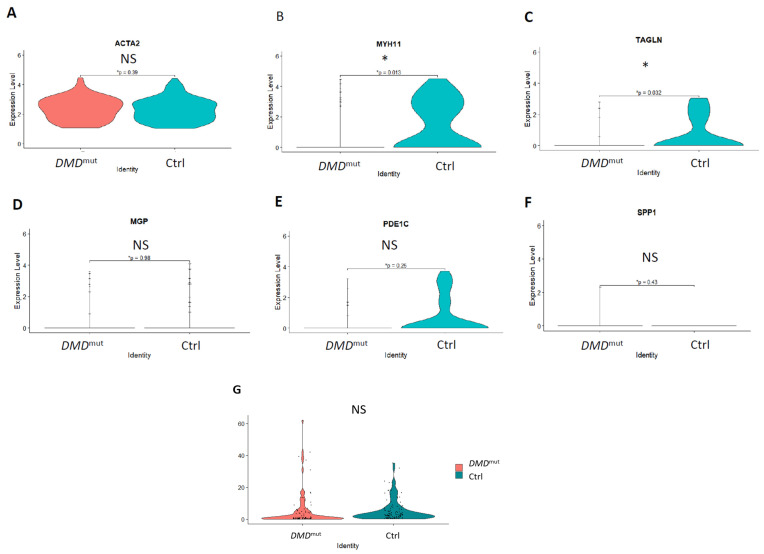
Violin plot illustrations of the contractile and synthetic gene marker analyses for ACTA2-annotated pan-VSMCs from *DMD*^mut^ and control muscles. (**A**) The expression of ACTA2 gene between VSMCs from individual cells with *DMD* mutations and control muscles. (**B**,**C**) The expression of contractile gene markers (MYH11 and TAGLN) between VSMCs from individual cells with *DMD* mutations and control muscles (* *p* < 0.05). (**D**–**F**) The expression of synthetic gene markers (MGP, PDE1C, and SPP1) between VSMCs from individual cells with *DMD* mutations and control muscles (NS: no significant difference). (**G**) The expression level of percentage of mitochondrial genes between VSMCs from individual cells with *DMD* mutations and control muscles (NS: no significant difference).

VSMCs can transform their phenotype from a contractile to a synthetic state. Therefore, it is intriguing to explore whether the loss of *DMD* expression could cause such a phenotypic transformation. In our study, we used Myh11 as a marker for the contractile phenotype of VSMCs. However, because VSMCs can also exhibit synthetic phenotypes, it is essential to consider both contractile and synthetic VSMC cell markers and to examine whether the *DMD* mutation affects the expression levels of these gene markers. Accordingly, α-smooth muscle actin (ACTA2) is a cytoskeletal protein expressed by both contractile and synthetic VSMCs. Therefore, we used ACTA2 as a pan-smooth muscle cell marker to re-cluster the VSMC population. We conducted a comparison between VSMCs from individual cells with *DMD* mutations and control muscles to examine the expression of contractile gene markers (MYH11 and Transgelin [TAGLN]) and synthetic gene markers, such as the matrix gla protein (MGP), phosphodiesterase 1C (PDE1C), and secreted phosphoprotein 1 (SPP1) [12]. Our results revealed that although there was no difference in ACTA2 expression between *DMD*^mut^ VSMCs and Control VSMCs (Figure 6A), *DMD*-mutated VSMCs had reduced expression of contractile genes (MYH11 and TAGLN) (Figure 6B,C), but there was no significant difference in the expression of synthetic genes (MGP, PDE1C, and SPP1) (Figure 6D–F) compared to control VSMCs.

To investigate whether *DMD*^mut^ VSMCs were more susceptible to membrane rupture, we used the percentage of mitochondrial genomic transcript reads as a biomarker and compared it between *DMD*^mut^ VSMCs and control VSMCs. Our analysis indicated a slight increase in the percentage of mitochondrial genome transcripts in *DMD*^mut^ VSMCs compared to control VSMCs, but there was no significant difference, as shown in Figure 6G. This increase in mitochondrial genomic transcripts is indicative of plasma membrane rupture [13,14].

## 4. Discussion

In this study, we compared blood pressure between mice with *DMD*^mut^ and their wide-type control and found that *DMD* mutant mice exhibited a significant decrease in systemic, diastolic, and mean blood pressure compared to wide-type mice. By analyzing published snRNA-seq datasets from the skeletal muscles of *DMD*^mut^ mice and control mice, we found that the most significantly activated pathways in *DMD*^mut^-VSMCs are involved in ion channel function. Notably, we discovered that the *DMD*^mut^-VSMCs showed significantly upregulated expression of KCNQ5 and RYR2. Moreover, snRNA-seq data analysis also indicated that *DMD* mutation altered major energy metabolism pathways in VSMCs, which included dysregulation of pyruvate metabolism and nuclear acid metabolism.

*DMD* is not only expressed in skeletal muscle cells and cardiomyocytes, but also in vascular smooth muscle cells (VSMCs) and non-vascular smooth muscle cells (non-VSMCs). PetKova M.V. et al. [15] recently generated a *DMD* (eGFP) reporter mouse through the in-frame insertion of the eGFP coding sequence behind the last *DMD* exon 79 and confirmed strong natural EGFP fluorescence at smooth muscle cells. In this study, we also confirmed the expression of *DMD* in vascular smooth muscle cells via immunofluorescent staining (Figure 1).

Recent reports have revealed that DMD patients often show low systolic blood pressure [16,17], and *DMD* patients with heart failure are also more susceptible to angiotensin receptor/neprilysin inhibitor with recurrent hypotension until discontinuation of this medication [6], suggesting that *DMD* might play a critical role in regulating blood pressure. However, a previous dog study [18], which generated some controversy, reported significantly increased systolic blood pressure in anesthetized dogs affected with X-linked muscular dystrophy (GRDM) after the administration of cisatracurium, a non-depolarizing neuromuscular blocking drug [18]. In this study, we compared blood pressure between *DMD*^mut^ and control mice, and found that *DMD*^mut^ mice exhibited lower blood pressure compared to their wide-type controls (Figure 2), which addressed the role of *DMD* in the regulation of blood pressure.

The role of *DMD* in the regulation of vascular smooth muscle cell phenotypes is largely unknown. *DMD* might regulate vessel response, as one report showed that DMD patients showed significantly higher intraoperative estimated blood losses without platelet abnormalities, which is independent of the duration of surgery, body weight, gender, and age. These might be attributable to impaired responsiveness of blood vessels [19]. *DMD* might also regulate VSMC proliferation in response to injury, and the expression of dystrophin in VSMCs may protect the artery wall against injury-induced intimal thickening, as one study revealed that mdx mice develop larger lesions with increased numbers of proliferating cells after a collar-induced injury of the carotid artery, suggesting that dystrophin deficiency stimulates neointima formation [20]. Recently, Lopez J.R. et al. [21] compared isolated VSMCs from *DMD*^mut^ and control mice and found a significant upregulation of TRPC1, -3, and -6 proteins in VSMCs isolated from *DMD*^mut^ mice compared to control mice. This study suggested that the lack of *DMD* in mutant VSMCs might produce a profound alteration of [Ca(2+)]i and [Na(+)]i homeostasis mediated by TRPC channels. *DMD* is one of the essential GPCR-interacting proteins for ensuring normal α(1D)-adrenergic receptor [α(1D)-AR] function in the regulation of vascular tone and blood pressure. Knock-out of multiple syntrophin isoforms resulted in the complete loss of α(1D)-AR function in mouse VSMCs and abrogation of α(1D)-AR-mediated increases in blood pressure [22].

It is critical to identify the key genes in the *DMD*-mediated regulatory networks of VSMCs, which is still largely unknown. To resolve this critical knowledge gap, we used unbiased snRNA-seq to identify *DMD*-related key genes in VMSCs and key regulatory pathways in VSMCs. Single-cell transcriptome sequencing is a state-of-the-art technology capable of dissecting cellular heterogeneity of transcriptomes of cell subpopulations at a single-cell resolution, thus providing new insight into the potential key genes and enriched pathways in VSMCs. SnRNA-seq analysis of muscles revealed that the most significantly activated pathways in *DMD*^mut^-VSMCs are involved in ion channel function (potassium channel activity, cation channel complex, and cation channel activity). Notably, we discovered that the *DMD*^mut^-VSMCs showed significant upregulation in the expression of KCNQ5 and RYR2.

This is a first-time report that *DMD* mutation increased KCNQ5 expression in VSMCs. KCNQ subfamily consists of five genes. Each encodes six transmembrane segments, including pore module S5–6 and voltage-sensing domain VSD, S1–4, which tetramerize to form a functional potassium channel [23]. KCNQ5 is expressed in VSMCs for normal regulation of vascular tone [24,25]. In VSMCs, KCNQ5 forms heteromultimeric channels with KCNQ4 to control arterial tone at rest. Pharmacological blockade of these channels leads to vasoconstriction [26]. KCNQ5 was recently reported to be a hypertension-associated gene in the mesenteric arteries of spontaneously hypertensive rats [27]. KCNQ5-selective activation has been reported to lower blood pressure by its vasodilatory action. Activation of voltage-gated KCNQ5 K(+) channels by Celecoxib has been reported to reduce the excitability and contractility of VSMCs [28]. Aloperine, a component of Sophora flavescens and an activator of KCNQ5, has been reported to relax mesenteric-resistant arteries and attenuate hypertension-associated vascular remodeling [29,30].

A previous study has reported that *DMD* mutation in VSMCs increases RyR2 activity [31]. RyR2 has important functions in systemic blood pressure control and adaptive vascular responses to pressure [9]. Targeted deletion of RyR2 in VSMCs has been reported to eliminate Ca^2+^ sparks, leading to increased myogenic tone and elevated systemic blood pressure [32,33]. Pritchard et al. [31] recently studied the effects of *DMD* mutation on VSMC contractility and found that the frequency and signal mass of spontaneous and transient Ca^2+^-release events through SR RyR2s (“Ca^2+^ sparks”) were greater in VSMCs from *DMD*^mut^ mice. Patch-clamp electrophysiological recordings indicated a corresponding increase in Ca^2+^-dependent BK channel activity, suggesting that increased size of RyR2 protein clusters in VSMCs from *DMD*^mut^ mice increases Ca^2+^ spark and BK channel activity, resulting in cerebral microvascular dysfunction. Pritchard H.A. et al [31] studied the effect of *DMD* mutations on the regulation of contractility in cerebral arterial and arteriovenous smooth muscle cells using *DMD*^mut^ mice. They reported that cerebral pial arteries and parenchymal arterioles from *DMD*^mut^ mice failed to produce appreciable spontaneous myogenic tone, and suggested that enhanced RyR and BK channel activity may be responsible for a decrease in pressure-induced constriction of arteries and arterioles of *DMD*^mut^ mice.

*DMD* is also expressed in non-vascular smooth muscle cells and plays an essential role in their function. Alves G.A. et al. [34] found that the ileum of *DMD*^mut^ mice showed a reduction in ileal muscular layer thickness and mild impairment of electromechanical and pharmacomechanical signaling associated with altered contraction induced by calcium influx. Furthermore, Yu H.H. et al. [35] recently generated *DMD*-mutated piglets using the CRISPR/Cas9 system and found that *DMD*-mutated piglets exhibited declining smooth muscle thickness in the stomach and intestine. Patients with muscular dystrophy exhibit gastrointestinal syndromes, such as hypomotility, pseudo-obstruction, and constipation [36,37]. To investigate the cause of smooth muscle contractility in *DMD* patients, Singh K. et al. [38] studied the expression of contractile proteins and the function of the colon in the colonic smooth muscles of *DMD*^mut^ mice. They observed decreased mRNA expression of contractile proteins in colonic smooth muscles of *DMD*^mut^ mice compared to control mice. They also found that contraction in response to acetylcholine and potassium chloride (KCl) was also decreased in colonic muscle strips and in isolated muscle cells of *DMD*^mut^ mice in comparison to control mice, indicating that *DMD* deficiency resulted in decreased contractility of colonic smooth muscles. *DMD* expression was also found in smooth muscle cells of urinary bladders, which might be related to the micturition problem in *DMD* patients [39,40]. Overall, these findings confirm the importance of *DMD* in the contractility of non-vascular smooth muscle cells for the maintenance of normal GI and urinary bladder function.

## 5. Limitations

To eliminate batch effects in our analysis of smooth muscle cells, we used the Harmony algorithm. In addition, the absence of functional dystrophin in the muscle cells leads to membrane damage [41]. To ensure cell viability, we filtered out cells with a high percentage of mitochondrial genomic transcripts, which may indicate cell membrane rupture. However, we recognize that a more rigorous quantitative approach may be required to fully comprehend the effects of these factors on our interpretation. Moreover, there are limitations to utilizing the existing snRNA-seq dataset for skeletal muscle analysis. Specifically, the snRNA-seq dataset we evaluated came from TA muscles of C57/BL6N mice, which were WT and *DMD* Exon 51 Knockout mice subjected to the CRISPR-Cas9 system, whereas our blood pressure and immunostaining data originated from DBA/2 mice with WT and *DMD* Exon 23 mutation.

## 6. Conclusions

In summary, we found that *DMD*^mut^ mice had significantly lower systolic, diastolic, and mean blood pressure compared to control mice. By analyzing the snRNA-seq data from *DMD*^mut^ and control mice, we found that the major activation pathways in *DMD*^mut^-VSMCs are involved ion channel function, such as potassium channel activity, cation channel complex, and cation channel activity. We also found that *DMD* mutations significantly upregulated the expression of KCNQ5 and RYR2 genes in VSMCs. Our study opens up new possibilities for the treatment of systemic hypotension in muscular dystrophy patients with pharmacological inhibition of KCNQ5.

## Data Availability

The snRNA-seq data was downloaded from NCBI Gene Expression Omnibus (GEO) public database (GSE156498).
